# Mitochondrial metabolism determines the functional status of human sperm and correlates with semen parameters

**DOI:** 10.3389/fcell.2022.926684

**Published:** 2022-08-30

**Authors:** Pilar Irigoyen, Paula Pintos-Polasky, Lucia Rosa-Villagran, Maria Fernanda Skowronek, Adriana Cassina, Rossana Sapiro

**Affiliations:** ^1^ Departamento de Histología y Embriología, Facultad de Medicina, Universidad de la República, Montevideo, Uruguay; ^2^ Departamento de Bioquímica, Facultad de Medicina, Universidad de la República, Montevideo, Uruguay; ^3^ Centro de Investigaciones Biomédicas (CEINBIO), Facultad de Medicina, Universidad de la República, Montevideo, Uruguay

**Keywords:** mitochondria, sperm metabolism, antioxidant status, respirometry assay, reactive oxygen species, male fertility

## Abstract

The diagnosis of male infertility is based essentially on the patient’s medical history and a standard semen analysis. However, the latter rarely provides information on the causes of a possible infertility, emphasizing the need to extend the analysis of the sperm function. Mitochondrial function has been associated with sperm function and dysfunction, the latter primarily through the production of excessive amounts of reactive oxygen species (ROS). We hypothesized that analysis of sperm mitochondrial metabolism together with sperm ROS production could be an additional tool to improve routine semen analysis, after appropriate validations. To test our hypothesis, we performed several experiments using a non-routine method (high-resolution respirometry, HRR) to access mitochondrial function. First, we investigated whether mitochondrial function is related to human sperm motility and morphology. When mitochondrial metabolism was challenged, sperm motility decreased significantly. Additionally, morphological abnormalities in the sperm mid-piece and mitochondria were associated with global sperm defects evaluated by routine methods. Subsequently, sperm mitochondrial function was assessed by HRR. Respiratory control ratio (RCR) was determined and evaluated in the context of classical sperm analysis. In parallel, sperm hydrogen peroxide (H_2_O_2_) production and seminal plasma (SP) antioxidant capacity were measured. The percentage of sperm with progressive motility correlated positively with RCR, SP antioxidant capacity, and negatively with the concentration of extracellular H_2_O_2_ production ([H_2_O_2_]). The percentage of normal sperm morphology correlated positively with RCR and negatively with [H_2_O_2_]. Sperm morphology did not correlate with seminal plasma antioxidant capacity. Furthermore, Receiver Operating Characteristic curves were used for the first time to test the diagnostic ability of RCR, [H_2_O_2_], and SP antioxidant capacity as binary classifiers. An RCR cut off value of 3.2 was established with a sensitivity of 73% and a specificity of 61%, using reference values considered normal or abnormal in routine semen analysis. The cut off value for [H_2_O_2_] was 0.2 μM/10^6^ sperm (sensitivity = 65%, specificity = 60%). There were no reference values for SP antioxidant capacity that distinguished between abnormal and normal sperm samples. We conclude that sperm mitochondrial function indices in combination with [H_2_O_2_] may be useful tools to complement the routine semen analysis.

## Introduction

Infertility is a growing problem worldwide, affecting up to 15% of couples of childbearing age (Vander Borght and Wyns, 2018). Although the male factor is responsible for at least 30–40% of cases, the male contribution to infertility among couples has not traditionally been emphasized (Barratt et al., 2018; De Jonge and Barratt, 2019). Male infertility diagnosis is mainly based on the patient’s clinical history, clinical examination, and the analysis of standard semen parameters according to the World Health Organization guidelines, which rarely determines the causes or point to possible treatments (WHO, 2010; WHO, 2021; Barratt et al., 2022; Björndahl and Kirkman Brown, 2022). In some cases of male infertility altered parameters in the spermiogram have been detected during semen analysis, but even when endocrine, genetic, and biochemical laboratory tests are added to the male examination (Kliesch, 2014), no cause associated with male infertility is found. These cases are considered as idiopathic and may account for 30–40% of male infertility (Kliesch, 2014). This situation severely limits treatment strategies to rescue fertility, so the inclusion of laboratory advanced tests to the routine semen analysis may assist in accurately diagnosis of male infertility (Agarwal and Bui, 2017).

There are numerous data showing a correlation between semen parameters and both, mitochondrial morphology and function (Wilton et al., 1992; Gopalkrishnan et al., 1995; Mundy et al., 1995; Courtade et al., 1998; Marchetti et al., 2002; Gallon et al., 2006; Amaral and Ramalho-Santos, 2010; Pelliccione et al., 2011; Cassina et al., 2015; Uribe et al., 2017; Durairajanayagam et al., 2021; Tanga et al., 2021). Furthermore, fertilization rate might be related to the proportion of normal mitochondrial structure (Baccetti et al., 2002) and mitochondrial function has been associated to the ability of spermatozoa to fertilize oocytes in mice (Ferreira et al., 2021; Giaccagli et al., 2021) and in humans (Kasai et al., 2002; Malić Vončina et al., 2016; Boguenet et al., 2021).

Despite the large body of data pointing to the importance of mitochondria in sperm function, the mechanisms by which this organelle operates in the male gamete are not fully understood, mainly in what concerns metabolic aspects. Specifically, in many species, including mice (Miki et al., 2004; Mukai and Okuno 2004; Castellini et al., 2021) and humans (Nascimento et al., 2008), is claimed that glycolysis would be used as a preferential pathway to synthesize ATP for maintaining sperm motility. Yet, we and others have previously shown that mitochondrial function is associated to sperm motility in men (Marchetti et al., 2002; Gallon et al., 2006; Amaral and Ramalho-Santos, 2010; Cassina et al., 2015; Uribe et al., 2017), and that coupling efficiency (that reflects the ability to produce mitochondrial ATP) is associated to the cell’s ability to fertilize in mice (Ferreira et al., 2021). All in all, previous result, obtained independently, suggest that ATP produced by mitochondria can make an important contribution to sperm function. When producing ATP, in analogy to somatic cells, sperm mitochondria are the major source of reactive oxygen species (ROS) (Aitken et al., 2012b; Cassina et al., 2015). During cellular respiration, approximately 0.2% of the oxygen consumed is converted to superoxide anions through electron leakage from the mitochondrial electron transport chain (Boveris and Chance, 1973; Nohl et al., 2003; Quijano et al., 2007). In the mitochondrial matrix and intermembrane space, superoxide interacts with the antioxidant enzyme superoxide dismutase, which catalyzes the dismutation of superoxide to hydrogen peroxide (H_2_O_2_) (Messner and Imlay, 2002; Wang et al., 2018). The H_2_O_2_ diffuses through biological membranes and can be measured in the extracellular space (Cardoso et al., 2012; Wang et al., 2018). The O_2_^•−^ reacts quickly with NO to form peroxynitrite. Low levels of O_2_^•−^, H_2_O_2_ and peroxynitrite, modulate cellular functions, but when they are produced in excess cause nitro-oxidative damage (Quijano et al., 2007). Nitro-oxidative stress is associated with altered semen parameters and abnormalities in the process of fertilization and pregnancy (Twigg et al., 1998; Agarwal et al., 2014a; Walters et al., 2020). In addition, mitochondrial dysfunction is associated with an increase in nitro-oxidative damage in sperm and a decrease in sperm motility (Cassina et al., 2015).

Sperm lose most of their cytoplasm and its organelles during differentiation, including some of the cell’s antioxidant defenses. It is postulated that the lack of antioxidant enzymes in spermatozoa is compensated by a high antioxidant capacity of seminal plasma (SP) (Agarwal and Deepinder, 2009). However, in clinical practice, neither ROS nor SP antioxidant capacity are usually tested (Agarwal et al., 2021a; 2021b). This may be because there is no consensus on appropriate tests and their clinically relevant cut off values, as well as an absence of standardization of laboratory techniques (Roychoudhury et al., 2016; Agarwal et al., 2021a, 2021b).

Considering the available information, we hypothesized that the analysis of sperm metabolic status and mitochondrial ROS production could provide additional information to complement routine semen evaluation. To prove our hypothesis, we analyzed sperm mitochondrial function using high-resolution respirometry (HRR) in the context of the routine semen analysis. Also, we standardized an assay to measure sperm H_2_O_2_ production and the antioxidant capacity of SP. We found some cut off values of mitochondrial function and H_2_O_2_ production that reflect the reference values of semen parameters established by World Health Organization (WHO). These tests could eventually be integrated into the andrology clinic after proper validation.

## Material and methods

### Reagents and media

Chemicals (unless otherwise indicated) were purchased from Sigma-Aldrich Chemical Co. (St. Louis, MO, United States).

### Selection of subjects and ethical guidelines

For this study, men who presented to the Fertilab andrology clinic (Montevideo, Uruguay) for semen testing were recruited. Recruitment was twice weekly, from August 2018 to December 2021. Unless otherwise stated, samples for the different studies were randomly selected (see below).

Clinical examination of patients and diagnostic semen analysis were performed at the andrology clinic according to the methods of WHO (WHO, 2010). Samples were discarded if they had a white blood cell count >0.5 × 10^6^/ml or leukocytospermia (WHO, 2010). Sperm cultures were negative for microorganisms. Men who had presented to the clinic for examination as sperm donors (e.g., semen donors for assisted reproduction procedures), that had genital tract infections, varicocele or control after vasectomy were excluded from the study. Not all men were tested for chromosomal, genetic or hormonal anomalies. None of these criteria constituted exclusion criteria.

A total of 339 men who met the inclusion criteria were finally enrolled in this study and randomly distributed as follows: 17 normozoospermic men were included in motility studies and ATP measurements only, 48 semen samples showing different semen parameters were selected for microscopic morphometric analysis. Between them, 5 normozoospermic healthy men who had conceived at least one child at the time of the study and 7 infertile patients were selected for electron microscopic analysis. Inclusion criteria for classifying a man as infertile were: A clinical history of infertility diagnosed as idiopathic (with abnormal spermiogram) and the absence of a female factor in the couple.

The remaining 274 samples were divided into two fractions to analyze HRR and sperm H_2_O_2_ production when the number of cells was sufficient. Ninety-five SP (considering a wide range of motility and morphology) of these samples were reserved for measuring their antioxidant capacity. The classification criteria for the samples are shown in Supplementary Figure S1. This categorization of samples was independent of fertility status.

The Ethics Committee of the Facultad de Medicina de la Universidad de la República Montevideo, Uruguay approved the experimental protocol. Before sample collection, all participants signed an informed consent form. The laboratory personnel assured the anonymity of the participants without the involvement of the researchers.

### Semen evaluation

Semen samples were obtained after 3 days of sexual abstinence by masturbation in special sterile containers. After liquefaction at room temperature for 30 min, semen volume, viability, pH, and normal morphology were analyzed according to the WHO guidelines.

Two sperm counting chambers CELL -VU^®^ (Millennium Sciences, Inc., New York, United States) were loaded, and ten different fields per chamber were randomly examined using a Nikon microscope at 37°C. Concentration and motility parameters were analyzed using an SCA-Microoptics automated analyzer (CASA) (Barcelona, Spain) with default settings according to WHO criteria (WHO, 2010).

### Preparation of the samples

Liquefied samples were centrifuged at 400 g for 10 min at room temperature to separate sperm from SP. If a subsequent washing step was included is described below. The SP was aliquoted and frozen at −80°C. Sperm were suspended in either of the following media: Biggers Whitten Whittingham medium (BWW: 95 mM NaCl, 4.6 mM KCl, 1.7 mM CaCl_2_, 1.2 mM KH_2_PO_4_, 1.2 mM MgSO_4_, 5.6 mM glucose, 0.27 mM sodium pyruvate, 20 mM acid-free HEPES, 25 mM NaHCO_3_, 44 mM lactic acid and 0.3% BSA, pH 7.4) for motility, morphology and respiratory experiments (Koppers et al., 2008), HAM-F10 medium (126 mM NaCl, 3.8 mM KCl, 1.08 mM NaH_2_PO_4_, 0.6 mM MgSO_4_.7H_2_O, 0.3 mM CaCl_2_, 6 mM glucose, 20 mM HEPES and 6 mM NaHCO, pH 7.4) for [H_2_O_2_] measurement or mitochondrial respiration medium (MRM: 0.1% BSA, 5 mM KH_2_PO_4_, 1 mM EGTA, 5 mM MOPS and 300 mM sucrose, pH 7.4) for motility studies and ATP analysis.

### Motility studies on the modulation of glycolysis and mitochondrial respiration

Sperm cells (35 × 10^6^) were suspended in 1,500 µL MRM and divided into seven aliquots. Aliquots were incubated at 37°C with: 1) 0.5% ethanol (OH), 2) 25 mM sodium oxamate (OXA) 3) 100 mM 2-deoxy-D-glucose (2DG), 4) 2.5 μM of antimycin A (AA), 5) 5 μM carbonil cyanide- p-(tri-flouromethoxy) phenyl-hydrazone (FCCP), 6) 25 mM OXA and 2.5 μM AA, 7) 100 mM 2DG and 2.5 μM AA. After 30 min, motility was analyzed using CASA. Aliquots were then centrifuged at 500 g for 10 min. Pellets were resuspended in 500 µL BWW and incubated at 37°C for 180 min, and sperm motility was measured. This study was performed on 11 normozoospermic men.

### ATP detection

ATP content was determined using a commercial kit (Cat #700410; Cayman Chemical, Ann Arbor, MI, United States). Spermatozoa (1 × 10^6^) from 6 normozoospermic males were incubated at 37°C for 30 min in MRM with the same drugs used in the motility studies (OH, OXA, 2DG, AA, FCCP, OXA + AA, and 2DG + AA). The medium was removed by centrifugation (600 g for 10 min), and the sperm pellet was washed twice with PBS at 4°C. Spermatozoa were resuspended in 100 µL of ATP detection sample buffer (1X) at 4°C, homogenized by repeated pipetting, and stored at −20°C until use. On the day of measurement, samples were thawed, diluted 1:10 with ATP detection sample buffer (1X), and stored on ice. ATP detection standards were prepared and the assay was performed according to the manufacturer’s instructions. The luminescence signal was recorded using a luminometer plate reader (Lumistar Galaxy, BMG, LabTech). Each measurement was performed twice.

### Fluorescence evaluation of sperm mitochondrial morphology

Sperm cells from 36 sperm samples were incubated with 50 nM MitoTracker^®^ Red CMXRos - M7512 - (Invitrogen, Waltham, Massachusetts, United States) for 30 min at 37°C in BWW and then spread on a glass slide. The slides were fixed in a mixed solution of 4% w/v paraformaldehyde in 0.1 M phosphate buffer (PB) for 30 min and washed three times in PBS. Sperm nuclei were counterstained with DAPI (4′,6-diamidine-2′-phenylindole dihydrochloride). Slides were mounted and observed using a Nikon Eclipse E400 epifluorescent microscope with a 100X, 1.4 NA oil objective (excitation: *λ* = 488 nm and *λ* = 543 nm). Digital images of 50–80 spermatozoa from each individual were acquired and processed. Manual segmentation of sperm intermediate pieces was performed from the MitoTracker channel images using ImageJ/FIJI (Schindelin et al., 2012). Areas of interest (ROI) were defined semi-automatically. Only isolated and well-defined midsections were included in the analysis (Figure 2A). Width, length, and circumference were automatically calculated for each ROI. The mean ± standard error (SE) was calculated for each sample.

### Electron microscopy

Semen from 5 fertile control men and 7 infertile men was analyzed by transmission electron microscopy (TEM). After liquefaction, the semen samples were centrifuged at 400g, the supernatant was discarded, and the pellets were fixed in a mixed solution of 4% w/v paraformaldehyde in 0.1 M PB containing 2.5% v/v glutaraldehyde (pH 7.4). Each preparation was then rinsed in PB (pH 7.4), post-fixed in 1% osmium tetroxide (w/v) for 1 h, dehydrated, and embedded in Araldite resin. After polymerization at 58–60°C for 48 h, sections were made on an RMC MT -X ultramicrotome using a DIATOME diamond knife. Semi-thin sections were prepared, stained with toluidine blue 1% w/v and examined under a Nikon ECLIPSE E200 light microscope. Adjacent ultrathin sections (50–70 nm) were stained with uranyl acetate followed by lead citrate and examined using a JEOL JEM-1010 transmission electron microscope at 80 kV accelerating voltage. Images were captured with a Hamamatsu C-4742-95 digital camera and processed with Photoimpact^®^ (Skowronek et al., 2012).

### Evaluation of sperm respiration control ratio by high-resolution respirometry

We analyzed mitochondrial function of spermatozoa from 171 men as previously described (Cassina et al., 2015). Oxygen consumption rate (OCR) of 30 × 10^6^ sperm resuspended in BWW was measured by high-resolution respirometry. HRR integrates highly sensitive oxygraphs (Oxygraph-2 K; Oroboros Instruments GmbH, Innsbruck, Austria) with software (DatLab, version 4.2; Oroboros Instruments GmbH) that displays respiration in terms of oxygen flux (pmol O_2_/1 × 10^6^ cells/sec). For all experiments, the stirrer speed was set to 750 rpm and the temperature was set to 37°C. Basal respiration was measured for 5–10 min before adding 2 µg/ml oligomycin, an ATP synthetase inhibitor. Maximal respiration was achieved by subsequent stepwise addition of the uncoupling agent 0.1–1 µM FCCP. Finally, a complex III inhibitor, 2.5 μM AA, was added to distinguish mitochondrial from residual oxygen consumption (non-mitochondrial respiration). Three indices are described by HRR: Coupling efficiency, spare respiratory capacity, and respiratory control ratio (RCR). We calculated: coupling efficiency (ratio between respiration associated with ATP synthesis and basal respiration), spare respiratory capacity (ratio between maximal and basal respiration rates), and finally respiratory control ratio (RCR, the ratio between maximal and oligomycin-resistant respiration rates) (Brand and Nicholls, 2011; Ferreira et al., 2021).

### Evaluation of sperm of hydrogen peroxide production

Hydrogen peroxide was measured using 10-acetyl-3,7-dihydroxyphenoxazine (Amplex Red). Amplex Red reacts in the presence horseradish peroxidase (HRP) with H_2_O_2_ in a 1:1 (v:v) stoichiometry to produce a highly red-fluorescent oxidation product, resorufin (Zhou et al., 1997; Messner and Imlay, 2002; González-Perilli et al., 2013). Amplex Red was dissolved in dimethyl sulfoxide (DMSO) to give a concentration of 1.25 mg/ml. This stock was frozen in aliquots and stored at −70°C for several months. A 0.8 ml aliquot of the stock was mixed with 18 ml of 50 mM potassium phosphate, pH 7.4, and kept shielded from light; this preparation is good for several hours (Messner and Imlay, 2002).

During standardization of the method a time-lapse analysis with and without 2.5 μM AA was done during 50 min (Figure 3A). Preliminary experiments were performed to determine the proper amount of human sperm cells and incubation time for H_2_O_2_ determination (data not shown). Sperm from samples of 136 males were washed with PBS by centrifugation at 400 g and re-suspended in HAM-F10. Between 2 and 6 × 10^6^ spermatozoa in HAM-F10 were placed in 96-well Nunc F plates in a final volume of 100 µL and exposed to HRP 8 μg/ml and Amplex Red (Invitrogen, Waltham, Massachusetts, United States) 50 μM. The H_2_O_2_ present in the sample was compared to a calibration curve of H_2_O_2_ (final concentration 0.03–8 μM). The measurements were performed for 30 min at 37°C in a Flash Spectral Scanning Multimode Reader (Varioskan, Thermo Fisher Scientific, MA United States) at *λex* = 530 *nm* and *λem* = 590 *nm*. Duplicates were performed in samples and triplicates to perform the standard curve.

### Evaluation of the antioxidant status of SP

The antioxidant capacity of SP from 95 men was determined by ferric-xylenol orange assay (FOX: 100 µM xylenol orange, 250 µM Fe^2+^, 4 mM BHT, 25 mM H_2_SO_4_, and 90% (v/v) methanol) (Jiang et al., 1990; Reyes et al., 2011) following the H_2_O_2_ consumption after 30 min (Gay and Gebicki, 2002). H_2_O_2_ oxidizes ferrous ions to ferric ions. To measure the ferric ions formed, we mixed 35 µL of plasma in a final volume of 1 ml of 50 mM phosphate-sodium buffer, pH 7.4, containing 100 µM H_2_O_2_. Aliquots of 100 µL were taken from the reaction tube and mixed with 900 µL of FOX at time points 0 and 30 min. All tubes were centrifuged at 2000 g for 5 min to remove all residual precipitate. H_2_O_2_ consumption was monitored by tracking the changes in absorbance at 560 nm at 25°C using a UV-Visible spectrophotometer UV-2450, Shimadzu. The H_2_O_2_ extinction coefficient was determined as 53.900 M^−1^cm^−1^. To determine the final concentration of H_2_O_2_, a standard curve of [H_2_O_2_] (concentration 5–200 µM) was included in the assay. At least three independent measurements were performed for each sample. Proteins in SP were quantified using the Bradford method (Bradford, 1976). Note that the less residual hydrogen peroxide measured, the more antioxidant capacity SP has.

### Statistics

Statistical analysis was performed based on the GraphPad Prism statistical package version 8.0.1 for Windows, GraphPad Software, San Diego, California United States, www.graphpad.com, and the statistical software JASP Team (2020), JASP (version 0.140). Data were expressed by arithmetic means, the corresponding standard errors, medians, and the 25th and 75th percentiles. Normal distribution of the data was tested using the Shapiro-Wilk normality test, which revealed that most sperm parameter values were not normally distributed. The Pearson test or Spearman correlation test was used to determine the relationship between the parameters and mitochondrial measurements. Comparisons between means were made using either the ANOVA test (multiple groups) or Student’s t test or Mann-Whitney test as a function of the normal distribution of the data (two groups). Receiver operating characteristic curves (ROC) were used to show the ability of mitochondrial function, [H_2_O_2_] and/or antioxidants to discriminate between samples with progressive motility ≥ vs. < 32%, normal sperm morphology ≥ vs. < 4% or normal vs. abnormal spermiogram. Finally, cut off values were chosen to maximize the sum of sensitivity and specificity. Hypotheses were contrasted with two tails, and a *p*-value of less than 0.05 was considered statistically significant. For the experiments to determine the cut offs, the sample size was calculated with a confidence interval (CI) of 95%, a margin error of 5 and 10% of the population.

## Results

### Studied population

A total of 339 men aged 18–63 years were included in the study. The mean, standard deviation (SD), median, maximum and minimum values of the six main semen analysis parameters are listed in Table 1. These include sperm concentration, ejaculate volume, sperm viability, round cell concentration, percentage of normal morphology, and progressive motility. Experiments were performed in a total of 206 normozoospermic men and 133 men with abnormal semen parameters (17 asthenozoospermic, 19 teratozoospermic, 30 oligozoospermic, 10 oligo-asthenozoospermic, 8 terato-asthenozoospermic, 17 oligo-terato-asthenozoospermic, and 32 oligo-teratozoospermic males) (Kruger et al., 1988; WHO, 2010) (Supplementary Figure S1).

**TABLE 1:** Descriptive characteristics of participants’ sperm parameters. Data were obtained by analyzing the first spermiogram of 339 individuals who attended the andrology clinic. Max = maximum value. Min = minimum value *lower reference values (WHO, 2010).

Parameter	Mean	SD	Median	Max	Min	*Lower reference limit
Patient age (yr)	34.9	7.3	35.0	63.0	18.0	—
Volume (ml)	3.6	1.5	3.5	10.0	0.5	1.5
Sperm concentration (million/mL)	47.7	38.0	40.6	214.0	0.04	15
Sperm viability (%)	86.5	11.7	89.0	99.0	3.0	58
Normal sperm morphology (%)	7.7	4.3	7.0	28.0	0.0	4
Progressive motility (a+b) (%)	54.4	17.2	58.0	90.0	3.0	32
Round cells (million/ml)	0.7	2.0	0.2	3.0	0.001	1

### Changes in mitochondrial function are related to human sperm motility and morphology

Since the role of mitochondria in human sperm function has not been fully elucidated (Ruiz-Pesini and Eperez 2000; Marchetti et al., 2002; Miki et al., 2004; Gallon et al., 2006; Ruiz-Pesini et al., 2007; Ferramosca et al., 2008; Koppers et al., 2008; Nascimento et al., 2008; Ferramosca et al., 2012; Sousa et al., 2011; Goodson et al., 2012; Piomboni et al., 2012; Marques et al., 2014; Moscatelli et al., 2017; Ammar et al., 2020; reviewed in: Amaral et al., 2013; Boguenet et al., 2021; Castellini et al., 2021), we first analyzed the role of the glycolytic and oxidative phosphorylation system (OXPHOS) in sperm motility and ATP production, as well as mitochondrial morphology of sperm.

**FIGURE 1:**
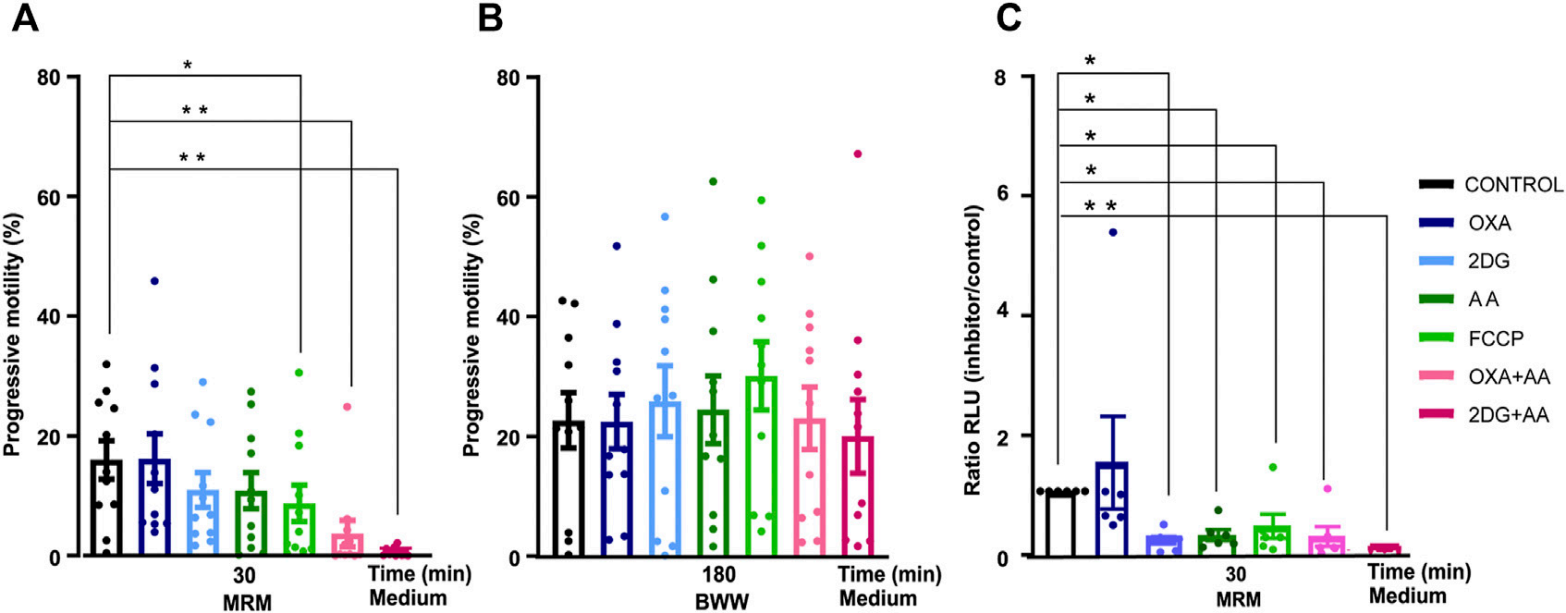
Changes in sperm motility and ATP production of human sperm incubated with metabolic inhibitors. Evaluation of the effects of glycolysis inhibitors and mitochondrial agents. **(A)** Progressive motility in presence of inhibitor and control at time = 30 min in MRM (*N* = 11) **(B)** time = 180 min in BWW (*N* = 11); **(C)** ATP production at 30 min in MRM medium (*N* = 6). Bars represent means ± SEM. Statistical significance between the control and the different groups was assessed with a ANOVA, Dunnett’s multiple comparison paired test *p < 0.05 **p < 0.01. OXA (oxamate) 2DG (2-deoxy-glucose), AA (antimycin A), FCCP (carbonil cyanide- p-(tri-flouromethoxy) phenyl-hydrazone), MRM (Mitochondrial Respiration Medium) and BWW (Biggers Whitten Whittingham).

First, we incubated spermatozoa from normozoospermic men in medium without nutrients (MRM) and added either glycolytic or mitochondrial function inhibitors. We used drugs that act at different levels of cellular metabolism: 2DG inhibits glycolysis by competing with endogenous glucose, OXA is an analog of pyruvate and inhibits the production of lactate, whereas FCCP is a respiratory chain uncoupler from the OXPHOS, and AA inhibits the mitochondrial complex III. Neither OXA nor 2DG nor AA significantly modified sperm motility. The sperm motility decreased significantly when the mitochondrial function was challenged by FCCP (Figure 1A). The combination of AA (mitochondrial inhibitor) and OXA or 2DG (glycolytic inhibitors) were required to completely decrease sperm motility. To test sperm viability, we washed sperm and incubated them in medium containing substrates (glucose, lactate, and pyruvate) for 3 h. Under these conditions, recovery of sperm motility was complete in all groups studied. (Figure 1B).

Second, we examined sperm ATP production under the same conditions as in the motility experiments. Sperm ATP production decreased under all the different conditions except when we added OXA (Figure 1C).

**FIGURE 2:**
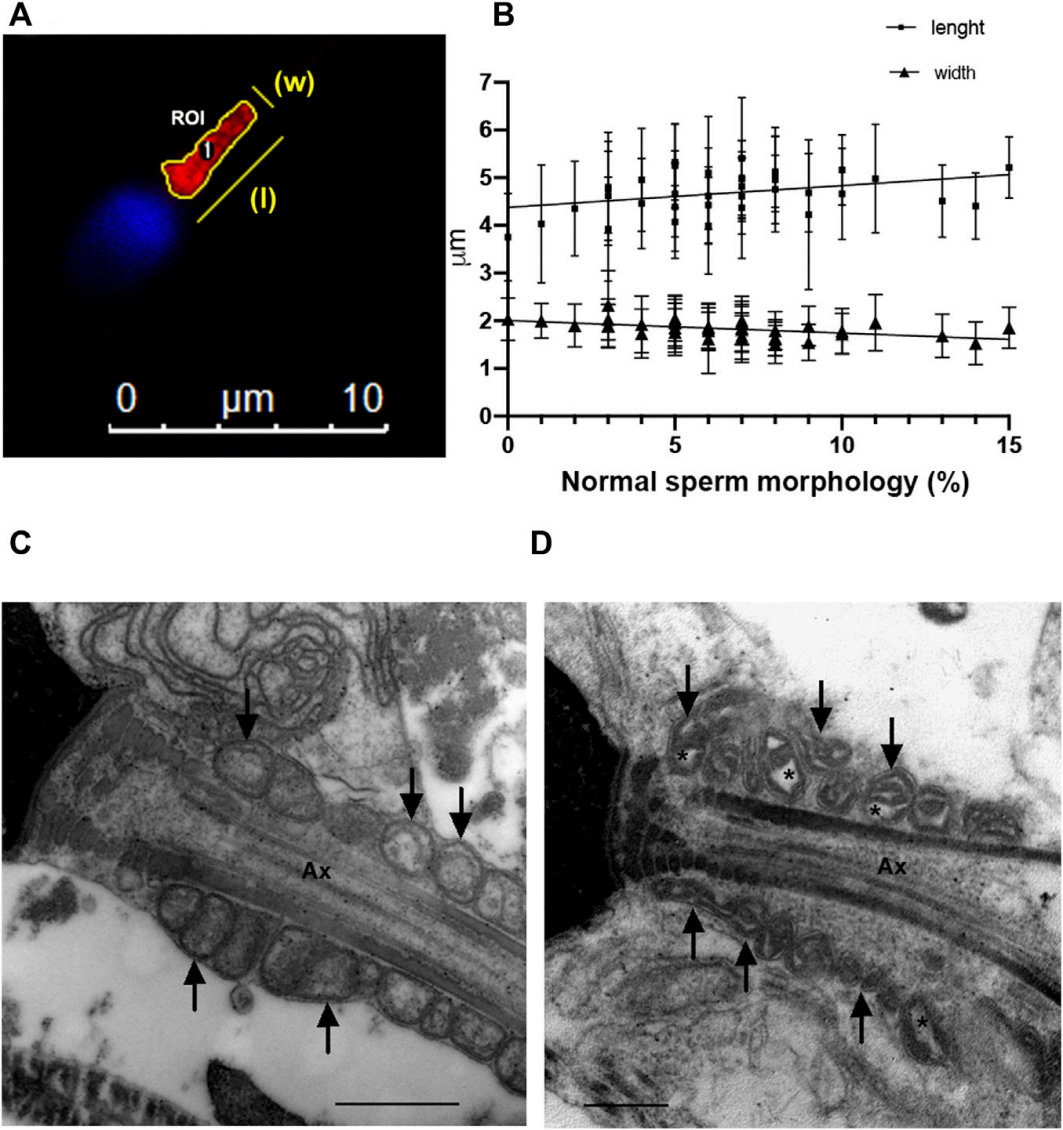
Sperm morphology analysis **(A)** Epifluorescence microscopy of the mid-piece of a human sperm cell. Manual segmentation of sperm mid-pieces using ImageJ/FIJI; **(B)** Correlation between mid-piece length and width and normal sperm morphology (*N* = 36). **(C)** Electron microscopy of sperm from a normozoospermic fertile man. **(D)** Electron microscopy of sperm from an infertile man with abnormal semen parameters. Defects on mitochondria are shown with arrows and asterisks indicate alterations in mitochondrial membranes. Ax = axoneme. Bars: 500 nm.

Finally, sperm mid-piece and mitochondrial morphology were analyzed (Figure 2). The sperm mid-piece was labeled with a red fluorescent dye that stains mitochondria (Figure 2A). Mid-piece length was positively correlated (Pearson correlation) with the percentage of normal morphology sperm in the semen samples (*r* = 0.37, *p* = 0.02), while mid-piece width had a negative correlation with normal progressive motility (graph not shown) and morphology parameters (Figure 2B) (*r* = −0.56 *p* = 0.0004 and *r* = −0.57 *p* = 0.0013, respectively).

In addition, we examined sperm mitochondria using electron microscopy (Figures 2C,D). We observed that in spermatozoa from samples with a percentage of normal spermatozoa <4%, the sperm mitochondria had abnormal shapes with dilatation of the inner mitochondrial matrix (asterisks in a representative image in Figure 2D).

Overall, these results indicate that sperm motility in normozoospermic males depends on mitochondrial function and that spermatozoa can maintain motility through the mitochondrial pathway. On the other hand, in men with altered seminal quality, the mid-piece and the mitochondria also showed abnormalities.

### The mitochondrial function of spermatozoa correlates with the sperm parameters of the classical spermiogram

Next, we decided to investigate whether mitochondrial function could be used as an index of sperm health. We measured the OCR of intact spermatozoa in real time, before and after the addition of inhibitors and FCCP.

We examined 171 sperm samples with HRR and analyzed the results in the context of the classical spermiogram (Tables 2, 3). We obtained three indices representing mitochondrial function and dysfunction (Cassina et al., 2015; Ferreira et al., 2021). Respiratory indices are ratios of oxygen consumption. They represent proportions of two rates so they are internally normalized to cell number or protein mass (Brand and Nicholls, 2011). RCR correlated positively with progressive motility and the percentage of normal sperm morphology in semen samples (Table 2). Similarly, the coupling efficiency and the spare respiratory capacity correlated positively with the percentage of sperm that exhibited progressive motility. Coupling efficiency and spare respiratory capacity did not correlate with the percentage of normal sperm morphology. We also divided the three main indices into two groups according to the sperm parameters (WHO’s reference values) (Table 3). Only RCR showed statistically significant differences between samples in which both the percentage of motility and morphology were below or above the reference values. Mean ± S.D of RCR of samples with progressive sperm motility ≥32% were statistically higher than those with less than 32% (5.7 ± 6.8, *n* = 155, vs. 2.4 ± 0.9, *N* = 16, *p* < 0.001). Sperm from males with a percentage of normal morphology ≥4% have a higher RCR than those with abnormal sperm morphology <4% (5.4 ± 5.3, *N* = 150 vs. 3.7 ± 4.2 *N* = 11, mean ± SD, *p* = 0.03). Unexpectedly, and although viability correlated to RCR (*r* = 0.1754, *p* = 0.0266, Spearman correlation, graph not shown), we did not see statistically significant differences between samples in which the percentage of viable cells were below or above 58% (WHO’s reference value). Also, there were no differences when the indices were analyzed using the reference values for the other semen parameters analyzed (volume or concentration) (data not shown).

**TABLE 2:** Correlation between sperm parameters and mitochondrial indices. Coupling efficiency = ATP turnover/basal respiration. Spare respiratory capacity = maximal respiration rate/basal respiration. RCR = maximal respiration in the presence of FCCP/respiration in the presence of oligomicyn, *N* = number of donors. Statistical analysis was evaluated using Spearman’s test *p < 0.05, **p < 0.01, ***p < 0.001.

	Correlations					
	Progressive motility (%)			Normal sperm morphology (%)		
**Parameter**	***N***	**Spearmans’ r**	***p*-value**	***N***	**Spearmans’ r**	***p*-value**
Coupling efficiency	166	0.239	0.002**	144	0.146	0.08
Spare respiratory capacity	166	0.174	0.025*	144	0.050	0.540
RCR	171	0.303	<0.001***	147	0.276	<0.001***

**TABLE 3:** Main respiratory indices classified by sperm parameters. RCR = respiratory control ratio SD = standard deviation. Max = maximum. Min = minimum. Statistic analysis, Man Whitney test. *p < 0.05, ***p < 0.001.

Progressive motility											
Indices	Mean		*p*-value	SD		Median		Max		Min	
	≥32%	<32%		≥32%	<32%	≥32%	<32%	≥32%	<32%	≥32%	<32%
Coupling efficiency	0.510	0.442	0.684	0.187	0.161	0.504	0.412	0.955	0.800	0.045	0.200
Spare respiratory capacity	1.982	1.695	0.126	0.807	0.6866	1.788	1.610	6.570	3.267	0.521	0.635
RCR	5.658	2.400	<0.001***	6.796	0.914	3.550	2.124	57.231	4.19	0.744	0.857

Normal sperm morphology											
Indices	Mean		*p*-value	SD		Median		Max		Min	
	≥ 4%	<4%		≥ 4%	<4%	≥ 4%	<4%	≥ 4%	<4%	≥ 4%	<4%
Coupling efficiency	0.503	0.497	0.710	0.186	0.170	0.498	0.451	0.955	0.800	0.045	0.288
Spare respiratory capacity	1.992	1.694	0.350	0.826	0.552	1.814	1.726	6.570	2.413	0.521	0.635
RCR	5.434	3.746	0.032*	5.354	4.208	3.610	2.529	31.825	16.132	0.744	1.108

These results show that mitochondrial function is correlated with semen parameters. Moreover, RCR is the best index to discriminate between samples with normal and abnormal semen parameters.

### The percentage of sperm with progressive motility and normal sperm morphology correlates negatively with hydrogen peroxide production

First, we detected extracellular [H_2_O_2_] produced by sperm. For this purpose, we measured the reaction between Amplex red and H_2_O_2_ in the presence of HRP (Richer and Ford, 2001; Baumber et al., 2002; Yánez-Ortiz et al., 2021).

**FIGURE 3:**
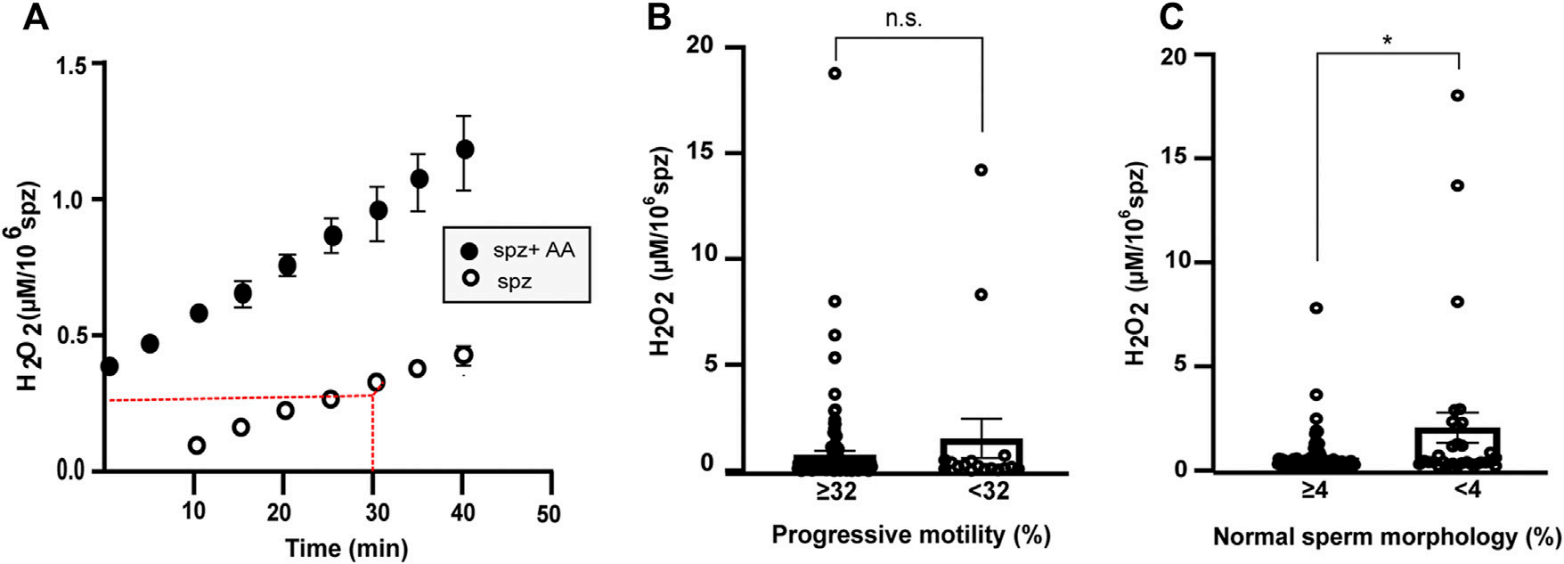
Production of extracellular H_2_O_2_ in s perm cells. **(A)** Production of peroxide by 10^6^ sperm cells (spz) from normozoospermic samples as a function of time using detection of resorufin fluorescence emission by Amplex Red assay. Comparison between sperm samples under control conditions (white) and in the presence of antimycin A (AA) (black). **(B,C)** Graphs show H_2_O_2_ concentration after 30 min in sperm samples classified by progressive motility cut off (≥32% *N* = 119; <32% *N* = 17) **(B)** or percentage of normal and abnormal sperm morphology (*N* = 104 and *N* = 31, respectively, cut off = 4) **(C)**. Error bars represent SEM. A Mann-Witney test was used to evaluate statistical significance. n.s. P > 0.05, *p < 0.05.

Upon addition of AA, an increase in H_2_O_2_ production is observed compared with baseline, suggesting that mitochondria may be a source of H_2_O_2_ in sperm cells as previously shown (Figure 3A) (Koppers et al., 2008; Storey, 2008; Aitken et al., 2012b; Escada-Rebelo et al., 2020).

Subsequently, 136 sperm samples with different sperm parameters were analyzed. The H_2_O_2_ production per million spermatozoa correlated negatively with sperm concentration (*r* = 0.32, *p* < 0.001, Spearman correlation), percentage of spermatozoa with progressive motility, and percentage of normal sperm morphology (Table 4).

**TABLE 4:** Correlation between semen parameters and [H_2_O_2_] or percentage of residual hydrogen peroxide (antioxidant capacity). Spearman’s test was used. *p < 0.05, **p < 0.01 ***p < 0.001. N = number of samples. ^#^percentage of sperm normal morphology.

	[H_2_O_2_] per million sperm (µM/10^6^ sperm)			Residual hydrogen peroxide (%)		
	*N*	Spearmans’ r	*p*-value	*N*	Spearmans’ r	*p*-value
Progressive motility (%)	136	−0.175	0.042*	95	−0.342	<0.001***
Sperm morphology^#^	128	−0.261	0.003**	95	0.014	0.890
[H_2_O_2_] per million sperm(µM)	—	—	—	45	0.080	0.500

There were no statistical differences between the [H_2_O_2_] produced by samples with progressive motility ≥ or <32% (0.75 ± 0.19 µM/10^6^ spermatozoa and 1.53 ± 0.92 µM/10^6^ spermatozoa, respectively, mean ± SEM, Figure 3B). The [H_2_O_2_] value was significantly higher in the samples with percentage of normal sperm morphology less than 4% (Figure 3C). The mean ± SEM of samples with more than 4% normal morphology was 0.42 ± 0.10 µM/10^6^ spermatozoa and 1.96 ± 0.76 µM/10^6^ spermatozoa in samples with morphology <4% (Figure 3C). There were no statistical differences in the other parameters analyzed.

Thus, measuring extracellular [H_2_O_2_] correlates with main semen parameters. The method discriminates between men with percentage of normal sperm morphology ≥4% and <4% in their spermiogram using strict criteria (Kruger et al., 1988).

### Seminal plasma antioxidant status correlates with sperm motility

To analyze whether the negative correlation of the production of [H_2_O_2_] with sperm parameters could be a consequence of variations in the antioxidant capacity of SP, we examined the percentage of residual H_2_O_2_ after addition of SP.

**FIGURE 4:**
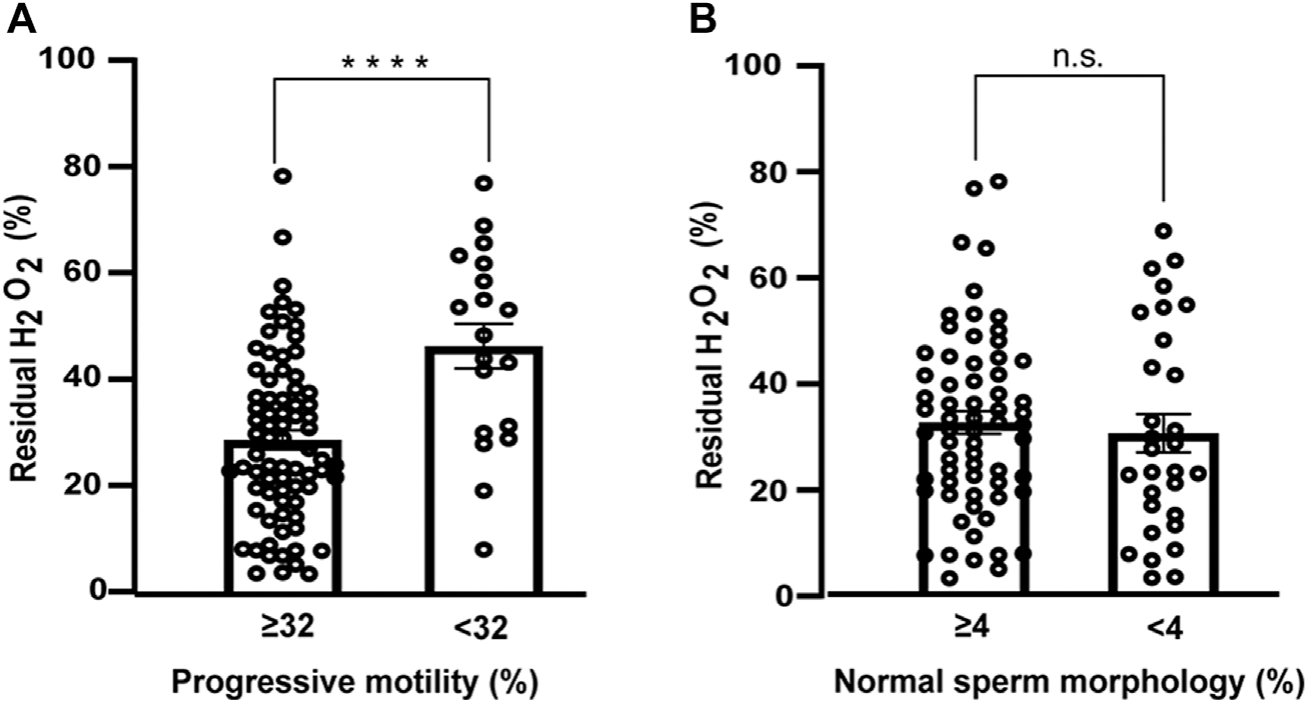
Antioxidant capacity of seminal plasma as a function of sperm parameters. **(A)** Progressive motility (≥32% *N* = 76; <32% *N* = 19) **(B)** normal sperm morphology (≥4% *N* = 65; <4% *N* = 30). Bars represent the percentage of residual H_2_O_2_ after 30min ±SEM. Mann-Whitney test was used to determine statistical significance n.s. P > 0.05, ****p < 0.0001.

The antioxidant capacity of SP correlated with sperm motility and was higher in samples with progressive motility ≥32% (Table 4 and Figure 4A). The mean ± SEM of the antioxidant capacity of SP was 28.6 ± 1.8% in men with progressive sperm motility ≥32% and 46.2 ± 4.2% in men presenting <32% *p* < 0.00001 (Mann Whitney test). The antioxidant status of SP did not correlate with the percentage of normal sperm morphology of the samples (Table 4 and Figure 4B). The means ± SEM were 32.7 ± 2.1% and 30.7 ± 3.6% when we analyzed SP of samples with normal sperm morphology of more and less than 4%, respectively (*p* = 0.4, *t*-test). The [H_2_O_2_] produced by the same sample did not correlate with the percentage of residual peroxide (Table 4). There were no differences when the indices were analyzed against the reference values for the other sperm parameters (data not shown).

These results suggest that the antioxidant capacity of SP has a strong correlation with progressive sperm motility. Samples with higher motility have higher antioxidant capacity, which is reflected in a lower percentage of residual peroxide. FOX method can distinguish between normal and abnormal sperm motility.

### RCR is a good indicator of the functional status of human sperm

**FIGURE 5:**
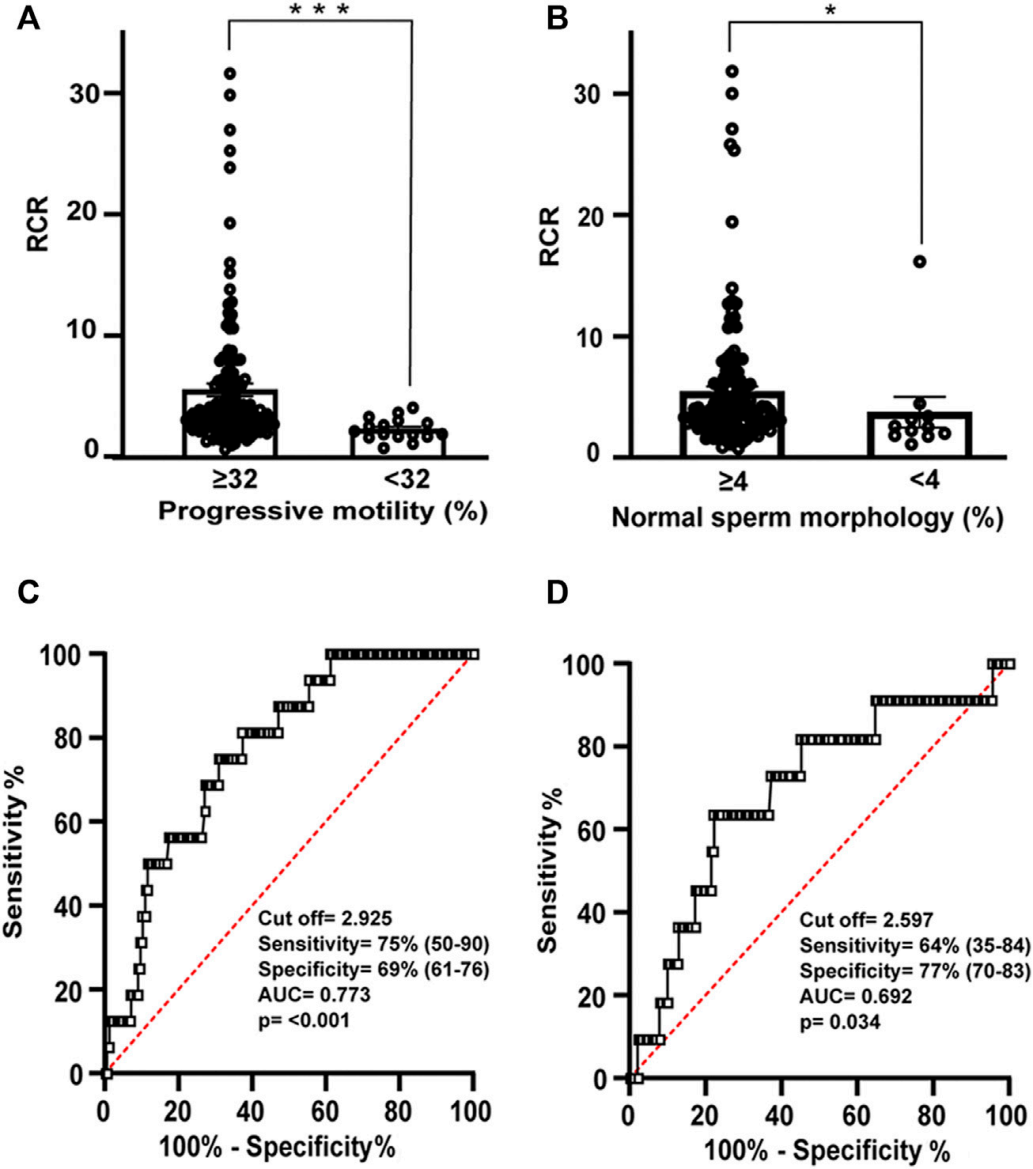
Relation between RCR, motility and morphology spermogram parameters. Bars represent means ± SEM of **(A)** progressive motility (≥32% *N* = 155; <32% *N* = 16) and **(B)** normal sperm morphology (≥4% *N* = 150; <4% *N* = 11). Mann-Whitney test was used to determine statistical significance; *p < 0.05, ***p < 0.001. **(C)** Receiver operating characteristic (ROC) curve showing RCR cut off value, sensitivity (%), specificity (%), and area under the curve for samples with normal or abnormal percentage of progressive motility and **(D)** ROC curve showing RCR cut off value, sensitivity (%), specificity (%), and area under the curve for samples with normal or abnormal percentage of spermatozoa with normal morphology.

The diagnostic ability of the previous indices as binary classifiers was determined using the ROC curves. The results were classified into two groups (using reference values of 32 and 4% for sperm motility and morphology, respectively) (Figures 5A,B; Table 3). The ROC curve showed good specificity and sensitivity in the case of RCR (Figures 5C,D). As for motility, the area under the curve (AUC) corresponded to 0.773 (95% CI between 0.672–0.873, *p* < 0.001). An RCR cut off point of 2.93 was established with a sensitivity of 75% and a specificity of 69% (Figures 5A,C). In the case of morphology, the AUC corresponded to 0.692 (95% CI between 0.524–0.860, *p* = 0.034) (Figures 5B,D). A cut off point of RCR of 2.60 was set with a sensitivity of 64% and a specificity of 77%.

A ROC curve (AUC = 0.651, 95% CI between 0.530–0.772, *p* = 0.012) determined 0.197 µM [H_2_O_2_]/10^6^ spermatozoa as the cut off value with 71% sensitivity and 61% specificity when samples were classified using references values of normal sperm ≥4% in their spermiogram (Figure 3C; Supplementary Figure S2). However, it was not possible to establish a cut off value of the [H_2_O_2_] when the reference value of the spermiogram was ≥32% of progressive motile sperm (Figure 3B).

In the case of residual hydrogen peroxide, a cut off value of 41% was set only when references value for motility were applied. The ROC curve has an AUC = 0.765 (95% CI between 0.539–0.892, *p* < 0.001) with 68% specificity and 79% sensitivity (Figure 4A; Supplementary Figure S3).

Thus, we were able to establish cut offs for the three different assays with respect to the classic spermiogram.

### RCR and H_2_O_2_ production can distinguish between normal and abnormal samples

Finally, to establish reference values encompassing all sperm parameters, we divided the male population into normozoospermic males and those with at least one abnormal sperm parameter (include: teratozoospermic, asthenozoospermic, oligozoospermic, terato-asthenozoospermic, and oligoasthenoteratozoospermic men).

**FIGURE 6:**
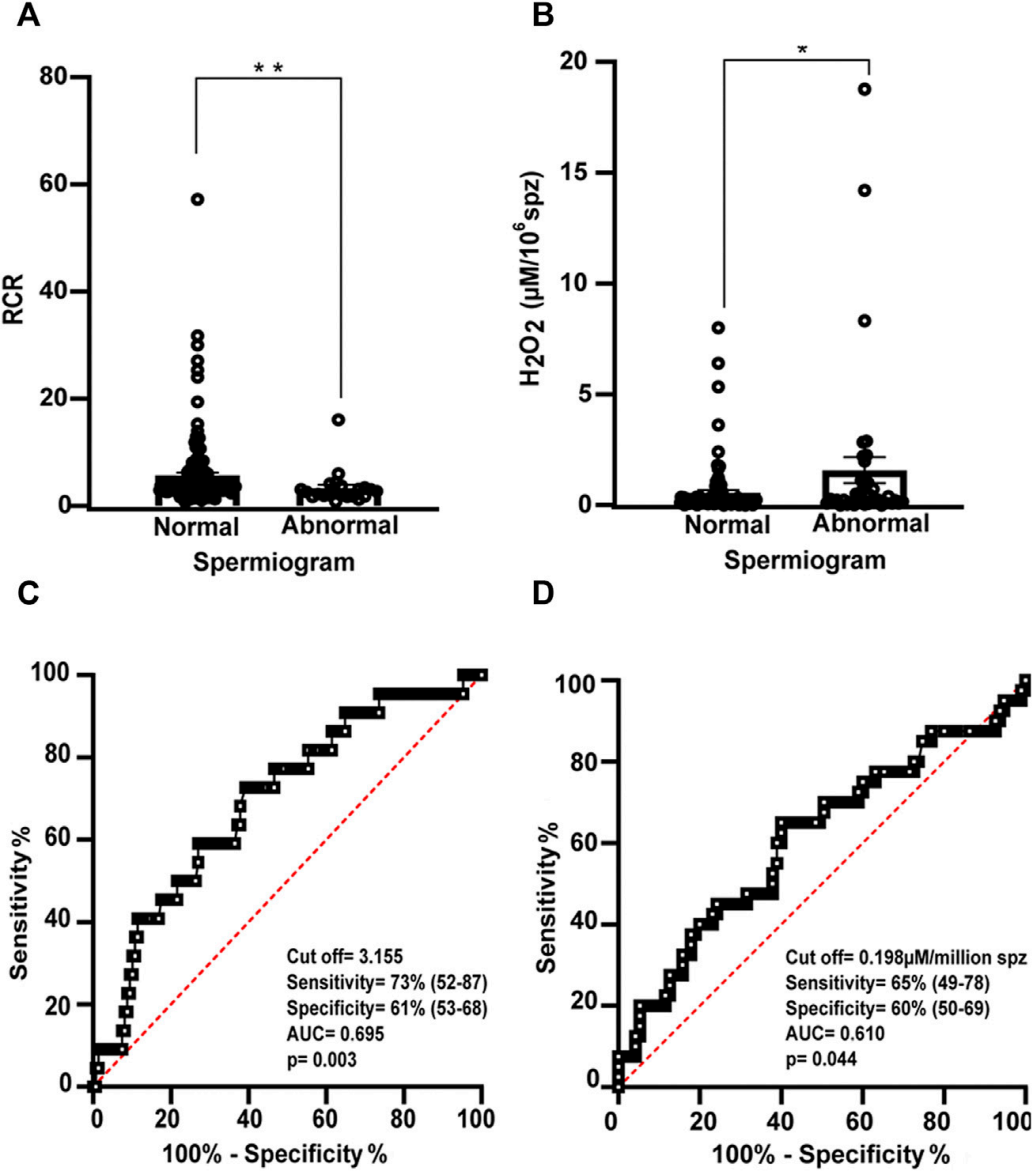
Relation between RCR and hydrogen peroxide concentration and spermogram parameters. Bars represent the mean values of **(A)** RCR (normal *N* = 148; abnormal *N* = 23) and **(B)** extracellular H_2_O_2_ concentration (normal *N* = 95; abnormal *N* = 40) ± SEM. A Mann-Witney test was used to evaluate statistical significance. *p < 0.05, **p < 0.01 **(C)** Receiver operating characteristic (ROC) curve showing RCR cut off value, sensitivity (%), specificity (%), and area under the curve for normal and abnormal spermiogram and **(D)** (ROC) curve showing [H_2_O_2_] cut off value (µM/million sperm), sensitivity (%), specificity (%), and area under the curve for abnormal and normal spermiogram.

The cut off value for RCR determined from the ROC curve (AUC = 0.695, 95% CI: 0.5792–0.810, *p* = 0.003) was 3.15 (sensitivity and specificity 73 and 61%, respectively) (Figures 6A,C).

The ROC curve (AUC = 0.610, 95% CI between 0.512–0.719, *p* = 0.044) determined a cut off value of [H_2_O_2_] = 0.198 µM/10^6^ spermatozooa (sensitivity of 65% and specificity of 60%) between both groups (Figures 6B,D).

When the population was divided into normozoospermic and abnormal spermiograms, no cut off value was determined for the percentage of residual hydrogen peroxide.

Therefore, RCR and [H_2_O_2_] are good methods for classifying semen samples with normal or abnormal spermiograms.

## Discussion

The source of ATP production for mammalian sperm motility has been widely studied (Miki et al., 2004; Mukai and Okuno, 2004; Miki, 2007; Ruiz-Pesini et al., 2007; Ferramosca et al., 2008, 2012; Goodson et al., 2012; Mukai and Travis, 2012; Piomboni et al., 2012; Moscatelli et al., 2017). The question of whether glycolysis or OXPHOS is the main source of ATP for sperm motility is controversial and considered species-specific (Storey, 2008; Amaral et al., 2013; Castellini et al., 2021; Kumar, 2022). By inhibiting the glycolytic pathway and mitochondrial function, we were able to confirm that energy supply (ATP) formed at the mitochondrial level is important for sperm motility in semen samples with normal parameters. Furthermore, we observed that motility and morphological alterations detected in routine semen analysis correlated with morphological changes in the mid-piece (Figure 2). Both observations make it reasonable to study mitochondrial sperm function in men with altered sperm parameters.

Our findings are consistent with some previous data emphasizing the role of OXPHOS as an essential ATP source for sperm function in humans and mice (Ruiz-Pesini et al., 1998; Ferramosca et al., 2008; Stendardi et al., 2011; Ferramosca et al., 2012; Piomboni et al., 2012; Ferramosca and Zara, 2014; Cassina et al., 2015; Moscatelli et al., 2017; Marín-Briggiler et al., 2021). Overall, it is now conceivable that both glycolysis and OXPHOS contribute to ATP production and depend on each other in controlling sperm functions reliant on the differential availability of energetic substrates in the environment (Castellini et al., 2021). There are also data from proteomic studies highlighting the contribution of lipid ß-oxidation as a sperm metabolic pathway (Amaral et al., 2013), suggesting that sperm metabolism is complex and needs further analysis.

We have previously shown that markers of mitochondrial function and dysfunction (such as RCR) reflect sperm activity (Cassina et al., 2015) and that they are associated with the cell’s ability to fertilize (Ferreira et al., 2021). The data presented here highlight the differences in mitochondrial function in men with different sperm parameters. We propose that assessment of mitochondrial function is useful to provide information on sperm metabolic status in men.

Several analyzes have associated semen parameters with mitochondrial function (Marchetti et al., 2002; Gallon et al., 2006; Amaral and Ramalho-Santos, 2010; Uribe et al., 2017; Durairajanayagam et al., 2021; Tanga et al., 2021). However, the evaluation of sperm mitochondrial activity is not usually part of human sperm testing studies. Oximetry and HRR in particular, have the advantage over other mitochondrial analyzes in that they can examine motile (live) and intact (non-permeabilized) spermatozoa (Cassina et al., 2015; Ferreira et al., 2021). In particular, RCR has high sensitivity to all points of mitochondrial function, so most of them can be analyzed in the same experiment, e.g., respiratory chain, mitochondrial membrane potential, and respiratory reserve capacity. It has the disadvantage of requiring expensive equipment and specialized personnel. Although the sensitivity of the equipment is very high, our team has determined that at least 12 million/ml of sperm are required to detect variations in oxygen consumption. We are aware that this low limit is not only a drawback of the method, but also a limitation of this study because we could not measure sperm from some oligozoospermic males. Other authors have used HRR to analyze sperm metabolism, looking for either changes in mitochondrial function under different experimental conditions (Cedikova et al., 2014; Hereng et al., 2014; Le Foll et al., 2021) or differences between sperm from normozoospermic and asthenozoospermic men (Cedikova et al., 2014; Cedíková et al., 2014) suggesting that this tool has potential applications.

Sperm oxygen consumption has been successfully measured using similar sensitive methods (e.g., Seahorse flux analyzer) (Tourmente et al., 2015; Balbach et al., 2020a, 2020b). These studies have demonstrated the importance of mitochondrial oxygen consumption in sperm capacitation in mice (Balbach et al., 2020b) and the differences between mitochondrial metabolism in several strains of mice (Tourmente et al., 2015). This last method has the advantage of analyzing extracellular acidification (ECAR) and oxygen consumption rates simultaneously. It can eventually be applied to a smaller number of cells. The disadvantage of Seahorse XF is that ECAR is based on measurements of extracellular pH near the plasma membrane, so these experiments exclude the use of HCO_3_^−^, which is required in some of the sperm media (Traba et al., 2016; Hidalgo et al., 2020). In the Seahorse flux analyzer, cells must adhere to the plates, potentially affecting metabolic functions. HRR has the property that cells can move freely, which is *a priori* an advantage in the case of sperm. Defects in sperm parameters are often directly related to high levels of ROS in the male reproductive tract and semen (Kumar et al., 2009; Agarwal et al., 2014b; Bonanno et al., 2016; Nowicka-Bauer and Nixon, 2020). Consistent with previous reports (Aitken et al., 2012b; Ammar et al., 2020), our data show an association between abnormal mitochondrial sperm morphology and altered sperm motility and morphology in subjects exhibiting multiple alterations in the spermiogram. In other cell types, mitochondrial structural changes have been reported as part of mitochondrial dynamics in response to ROS (Galloway et al., 2012; Martínez et al., 2019, 2020). In mature spermatozoa, mitochondria are arranged around flagella and form a thick mitochondrial sheath stabilized by di-sulfide bonds (Otani et al., 1988). This capsule-like structure provides mechanical stability, protection, and resistance to hypo-osmotic stress (Amaral et al., 2013). Therefore, sperm mitochondria are unlikely to undergo the same dynamic changes observed in other cell types. However, human sperm mitochondria exhibit a looser morphology during capacitation, likely due to an increase in mitochondrial volume (Vorup-Jensen et al., 1999). Capacitation is a functional change in sperm that prepares cells for fertilization (Austin, 1952) and requires small amounts (considered physiological) of ROS (de Lamirande et al., 1997). Whether increased ROS production can alter sperm mitochondrial morphology remains to be investigated.

All in all, these results argue in favor of studying mitochondrial analysis in parallel with the redox status of sperm and semen. Moreover, these results were also consistent with those of respirometry studies; when sperm parameters were abnormal, an increase in H_2_O_2_ was measured. In this study, we used a previously published assay (Richer and Ford, 2001; Baumber et al., 2002; Yánez-Ortiz et al., 2021) to detect H_2_O_2_ production in sperm.

We observed a negative correlation between sperm parameters and H_2_O_2_ production. These results are consistent with those obtained by other methods, such as a correlation between ROS production and oligozoospermia (Agarwal et al., 2014a), asthenozoospermia (Bonanno et al., 2016), and teratozoospermia (Agarwal et al., 2014b). It has already been reported that defective spermatozoa produce higher amounts of ROS than normal ones (Aitken et al., 2012b), but an effect of H_2_O_2_ upstream on spermatogenesis that interferes with the final sperm production and reduces sperm concentration and/or sperm motility and morphology cannot be excluded.

Although the sensitivity and specificity of various tests measuring ROS have been published, they remain variable, are not standardized, and generally cannot provide diagnostic recommendations (Aitken et al., 2012a; Benjamin et al., 2012; Sharma et al., 2017; Agarwal et al., 2021a). In addition, many tests for ROS are expensive, time-consuming, often require specialized training, and are therefore not readily available in clinical and diagnostic laboratories (Alahmar, 2019; Agarwal et al., 2021a; 2021b). Extracellular hydrogen peroxide measurement by amplex red has the limitation of being an indirect assay. However, this approach has several advantages over other ROS assays. As O_2_^•−^ does not cross membranes at physiological pH, measurement of hydrogen peroxide, which is the product of O_2_^•−^ dismutation and diffuses through membranes, can then be used as an indicator of mitochondrial O_2_^•−^ levels. Furthermore, peroxide is much more stable than O_2_^•−^ and can accumulate in easily detectable amounts (Messner and Imlay, 2002; Wang et al., 2018). Our results showed an increase in the formation of hydrogen peroxide when AA (inhibitor of the III complex of the respiratory chain) was added, confirming that mitochondria are a source of ROS formation. An advantage of measuring hydrogen peroxide by this method is that it does not require specialized technicians or sophisticated equipment. It also represents mitochondrial superoxide production in sperm (Wang et al., 2018).

SP contains many enzymatic and non-enzymatic antioxidants, which are a source of sperm protection against oxidative stress (Khosrowbeygi and Zarghami, 2007; du Plessis et al., 2008). Therefore, it is clinically relevant to establish the reference values for measuring ROS in combination with antioxidant capacity in SP (Agarwal et al., 2015). Using a sensitive method that quantifies the amount of residual hydrogen peroxide, we were able to determine the antioxidant capacity of several SP samples. There are already several essays available for measuring antioxidant capacity (Zini et al., 2000; Agarwal and Prabakaran, 2005; Baker and Aitken, 2005; Agarwal et al., 2006; Khosrowbeygi and Zarghami, 2007; Agarwal et al., 2015; Sharma et al., 2017; Robert et al., 2020). The reason for including this particular method in our analysis is that we can directly relate the production of ROS (H_2_O_2_) to the antioxidant assay chosen (the percentage of decay of H_2_O_2_ when de SP is added). In our study, seminal antioxidant capacity correlated positively with sperm motility. This is consistent with previous studies indicating that antioxidant capacity is reduced in asthenozoospermic men (Pahune et al., 2013; Madej et al., 2021). However, antioxidant capacity did not correlate with the percentage of normal sperm morphology. It is known that antioxidant capacity in SP is related to age, environmental pollution, lifestyle, chronic or genital (varicocele) diseases (Eroglu et al., 2014; Roychoudhury et al., 2016; Madej et al., 2021). The analysis of these factors was beyond the scope of our study, but some of them could explain the lack of correlation between the antioxidant capacity of SP and sperm morphological alterations in the spermiogram.

The measurement of oxidants in combination with the antioxidant capacity of a sample could be important to integrate the redox status of semen (Agarwal and Bui, 2017; Agarwal and Wang, 2017). Unexpectedly, in this work, the production of H_2_O_2_ and the antioxidant capacity of SP were analyzed in 45 samples, but no correlation between [H_2_O_2_] extracellular and the percentage of residual peroxide in SP of the same samples was found. We can speculate that either sperm ROS production is not related to the antioxidant capacity measured by this method, or that other factors may influence semen composition. Since environmental factors affect the antioxidant capacity of SP as suggested above, some of them may be studied in the future to test the latter hypothesis. Using a device that measures the relative proportions of oxidants to reductants (antioxidants) called Male Infertility Oxidative System (MiOXSYS) (Agarwal and Bui, 2017; Agarwal et al., 2021b), the oxidation-reduction potential (ORP) was determined. ORP correlated with sperm parameters and male fertility status (Agarwal et al., 2017), including data from a multicentric study (Agarwal et al., 2019). The technique is promising for the evaluation of sperm oxidative stress. The device is not yet available worldwide, but it will be an alternative for analysis of sperm samples in relation to sperm metabolism.

The present study was performed on samples that are not pure oligozoospermic, asthenozoospermic, or terathozoospermic. This is a limitation of the analysis, however, the main objective of this study was to establish cut off of mitochondrial function in parallel with spermiogram results. In particular, measurement of sperm RCR and their production of ROS by quantification of [H_2_O_2_] extracellular resulted in good indicators of sperm function and correlated with main semen parameters. Therefore, some reference values were determined. The data presented here show that RCR and [H_2_O_2_] are good binary classifiers; a RCR cut off point of 3.2 was established with a sensitivity of 73% and a specificity of 61% using reference values of 32 and 4% for sperm motility and morphology, respectively. The cut off value for [H_2_O_2_] was set at 0.2 μM per million sperm (sensitivity = 65%, specificity = 60%). In contrast, semen antioxidant capacity had not been demonstrated to be a good indicator for classifying semen samples with normal or abnormal spermiograms.

In this work, we established the cut off based on sperm parameters independently of the cause of the fertility status; e.g., we did not include the presence of chromosomal, genetic, hormonal abnormalities or other previously known conditions related to male fertility in the analysis. It will be interesting to test whether the reference values described can distinguish between these conditions.

Men who attend fertility clinics seek fertility advice, but not all men with abnormal sperm parameters are infertile and vice versa (Guzick et al., 2001; Cairo Consensus Workshop Group, 2020; Pandruvada et al., 2021). Infertility with normal semen parameters is one of the new challenges in this field. As a result, fertility clinics are looking for new indices to determine the true fertility potential of sperm. In our study, ROC curves were generated to determine the cut off values, sensitivity, and specificity of the assays using the previous reference parameters. We are aware that a weakness of the study is that the categorization is not between fertile and infertile men. Further studies are planned to apply the indices in the IVF clinic, where the true fertility potential of sperm can be assessed. In addition, multicentric validation of the results must be performed before these tools can be used in clinical practice. Nevertheless, the application of the mitochondrial and H_2_O_2_ sperm production indices may contribute to the understanding of sperm biology and may be helpful in explaining some cases of infertility.

## Conclusion

Semen evaluation is considered one of the most important laboratory tests for assessing male fertility. Although it is the gold standard method for studying infertile men, it still lacks functional analysis of sperm status. We propose that determination of RCR in combination with other analyzes (such as measurement of extracellular [H_2_O_2_] sperm production and antioxidant capacity of SP) provides results on sperm functional status focusing on energy metabolism, involvement of mitochondrial function, and formation of ROS.

## Data Availability

The raw data supporting the conclusion of this article will be made available by the authors, without undue reservation.
