# Supplementary figures and images for: Mitochondrial metabolism determines the functional status of human sperm and correlates with semen parameters
Source: Front Cell Dev Biol. 2022 Aug 30;10:926684. doi: 10.3389/fcell.2022.926684 (PMC9468643; doi:10.3389/fcell.2022.926684)

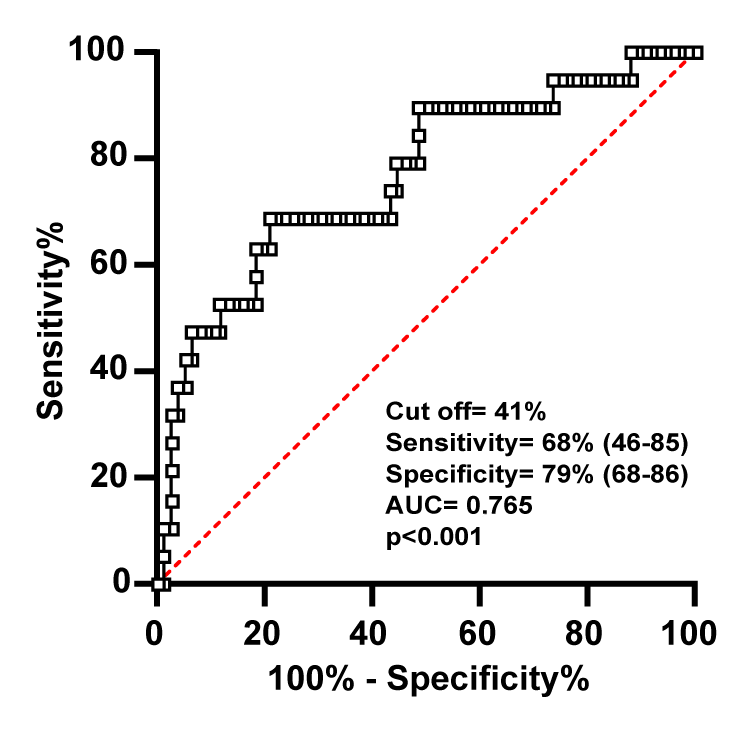

Supplement: Supplementary file 1 [file Image3.TIF]

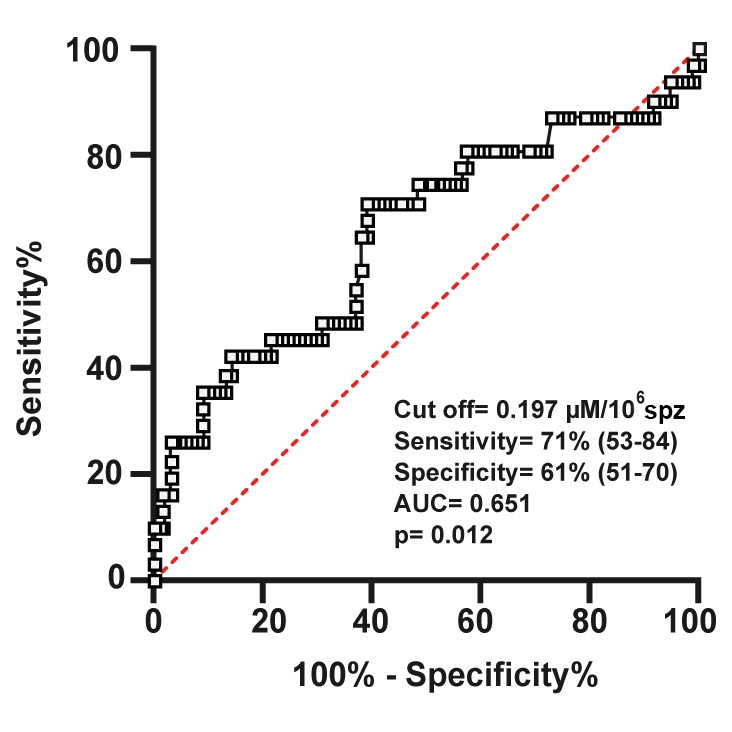

Supplement: Supplementary file 2 [file Image2.tif]

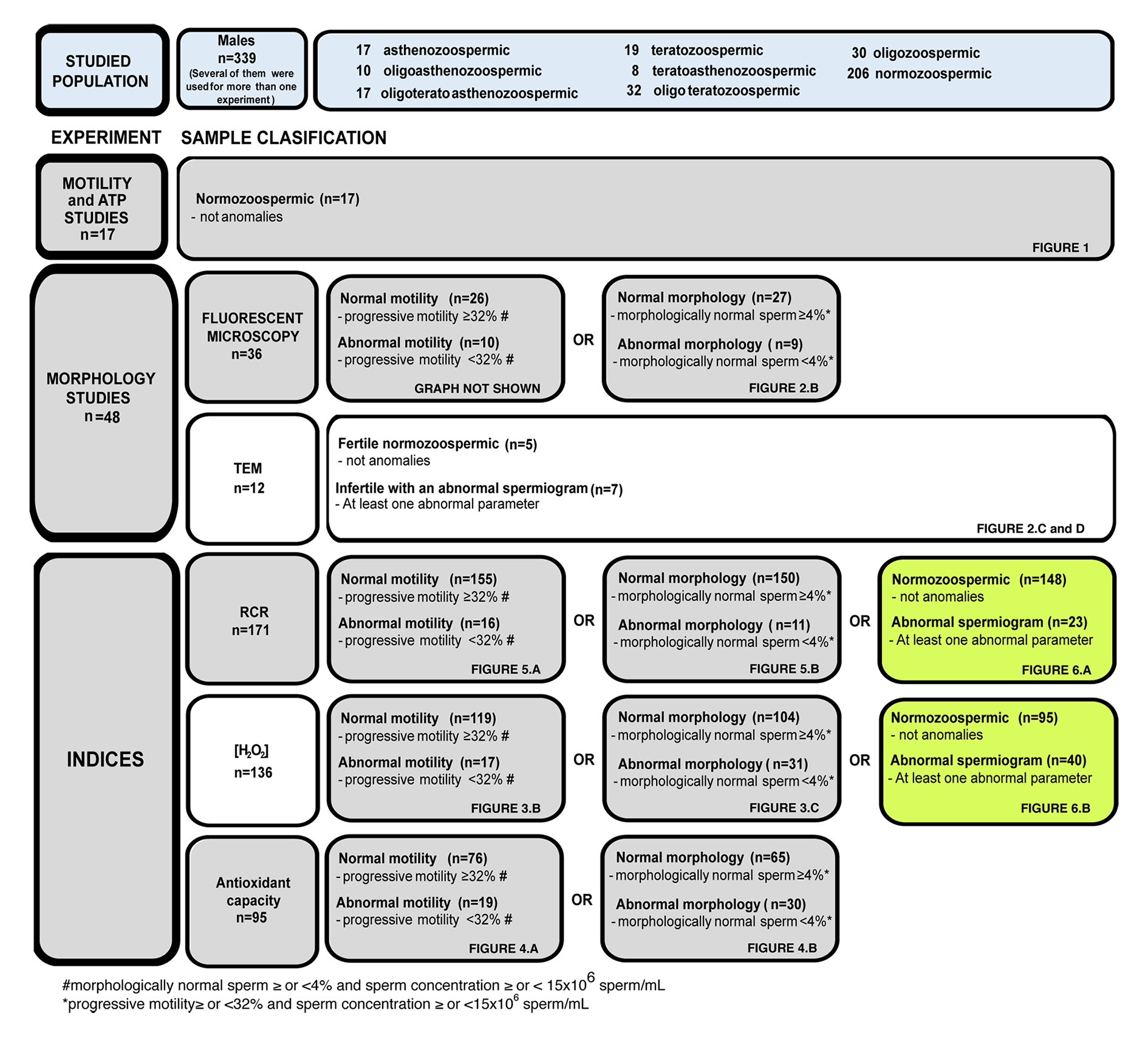

Supplement: Supplementary file 3 [file Image1.TIF]
